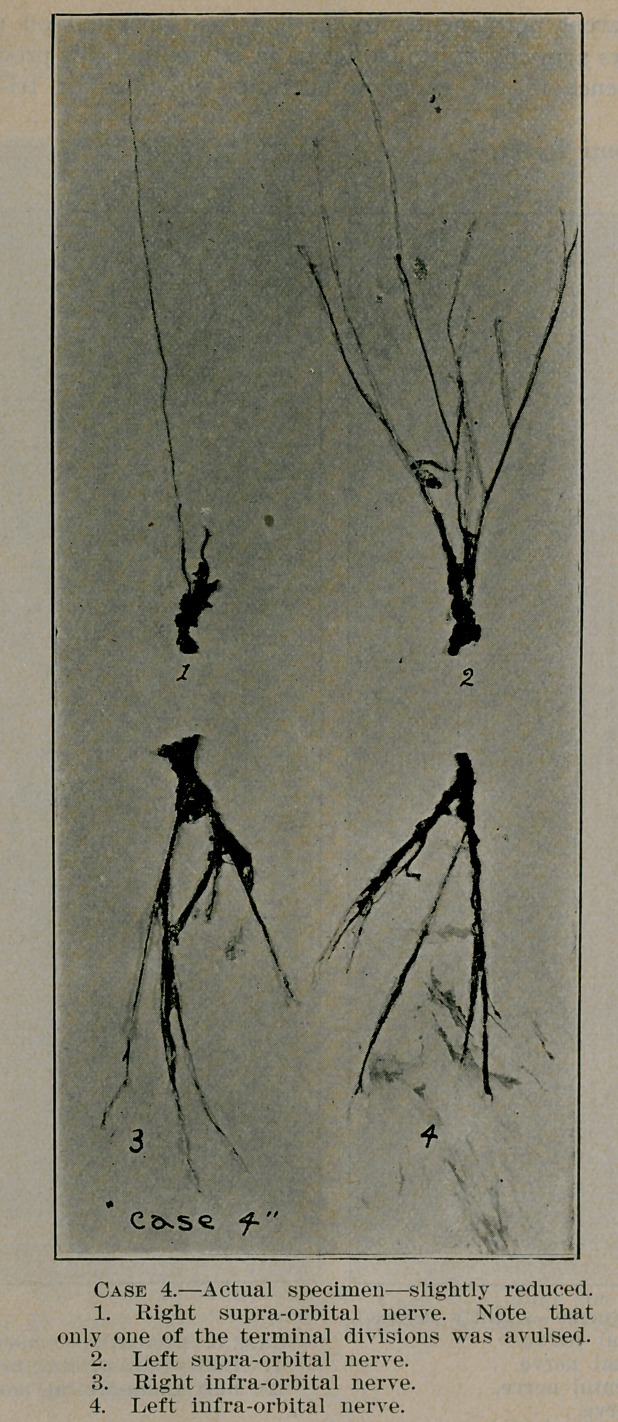# Avulsion of Nerves in Facial Neuralgia with Report of Four Cases

**Published:** 1913-07

**Authors:** 


					﻿ABSTRACTS.
Avulsion of Nerves in Facial Neuralgia With Report
of Four Cases, Herbert P. Cole, M. D., Mobile, Ala., Charlotte
Medical Journal, April, 1913. (Plates used by courtesy of
Editor.) Simple section of the nerve offers the least benefit
of the minor operations. The high mortality rate in resection
of the Gasserian ganglion always warrants our first attempting
minor procedures for the relief of this condition. Many cases
present grave lesions contra-indicating the radical Gasserian
ganglion operation. Of the Gasserian operations apparently
Murphy’s extra-dural section of the ganglion with the interposi-
tion of a wax plate pressed into the foramen ovale appears
to offer all the benefits of the Gasserian resection with a greatly
reduced mortality rate.
The permanency of cure from the avulsion method depends
not alone upon the thoroughness of the avulsion and whether
or not the ganglion itself has become involved, but also upon the
thoroughness with which we block the proximal nerve root to
prevent the regeneration so prone to occur in all sensory nerves.
The silver screw fixed in the infra orbital foramen offers a
successful obstacle to the regeneration of the infra-orbital divis-
ion. The supra-orbital stump has been successfully blocked by
rolling it in a flap of frontal periosteum which may be rolled
under the orbit. As yet we are unfamiliar with any method of
blocking the roots of the inferior dental or lingual nerves.
Conclusion.—The temporary relief obtained in all of these
cases in periods from seven to thirty-three months, together
with apparent permanent cure in one case operated on nearly
three years ago, certainly justifies us in attempting this procedure
in preference to the ganglion operation in cases of tri-facial
neuralgia.
202 Conti street.
				

## Figures and Tables

**Figure f1:**
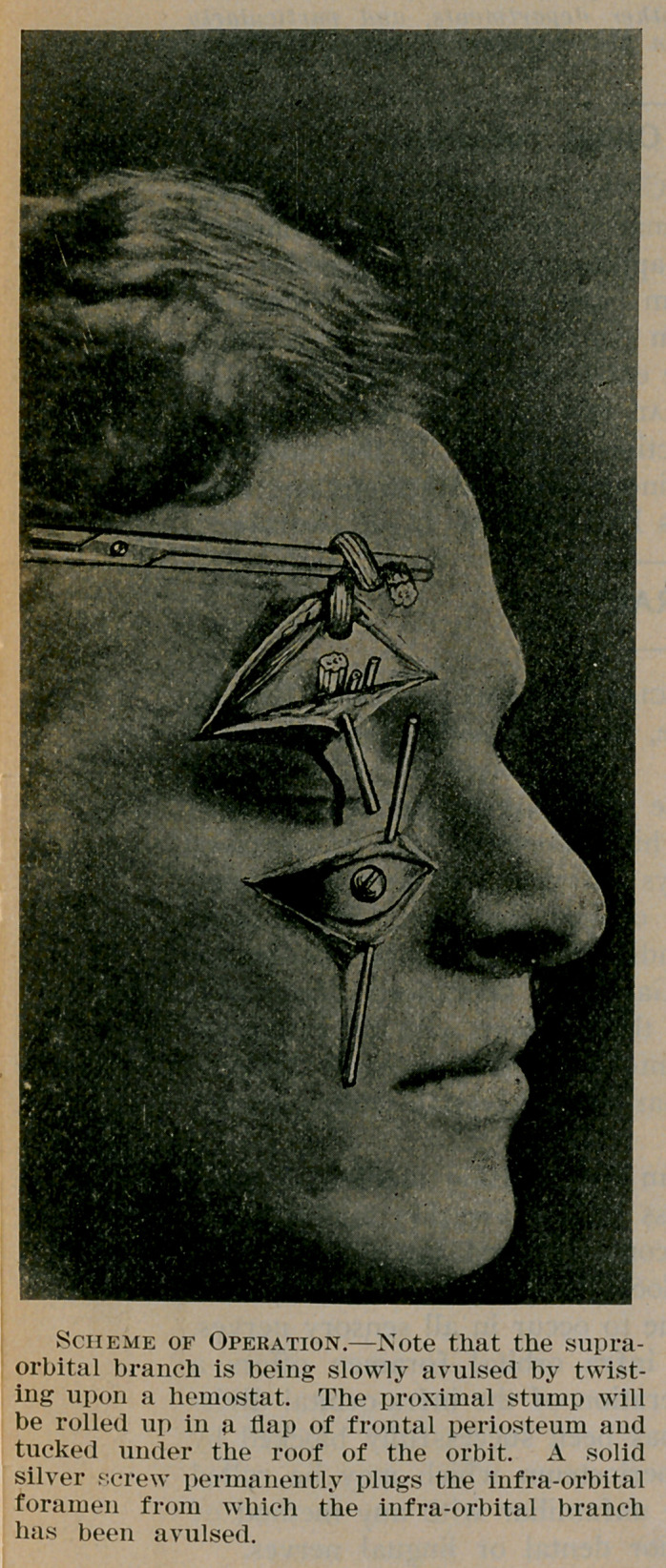


**Case 1. f2:**
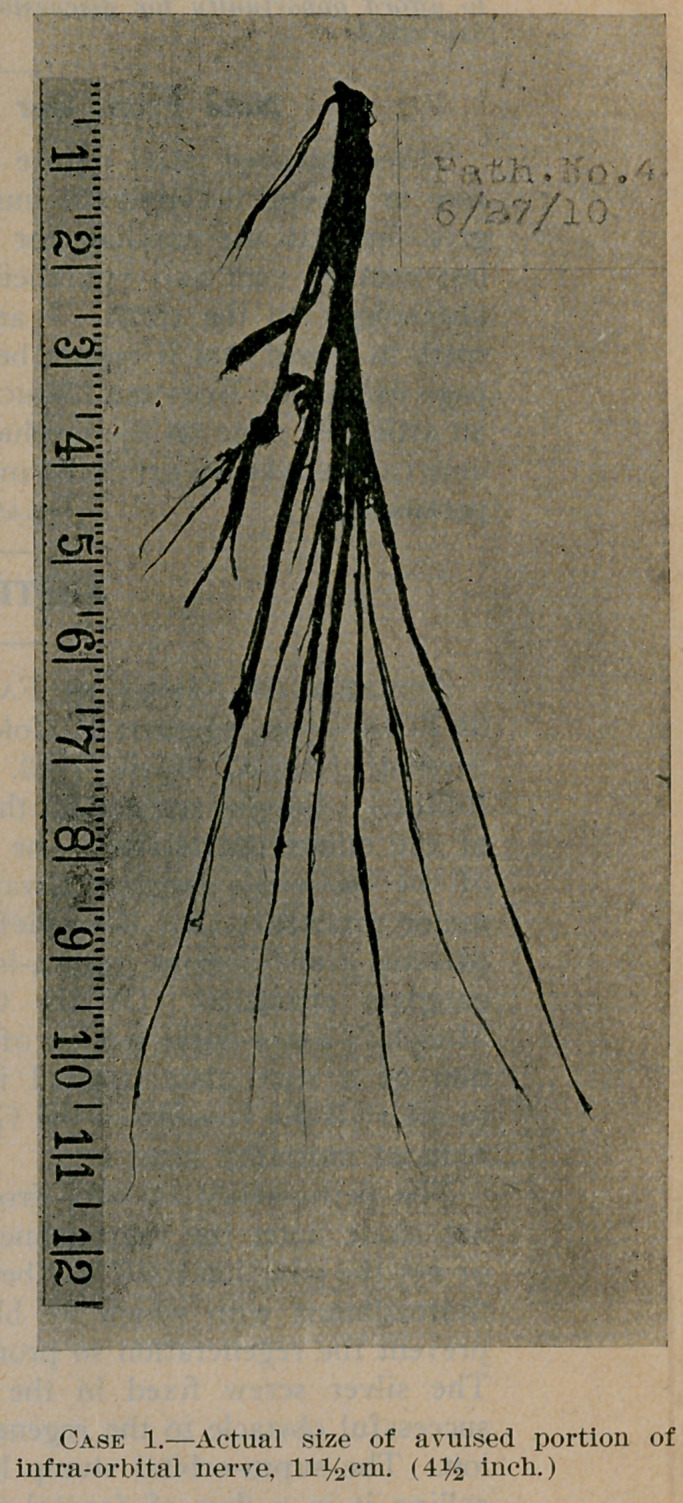


**Case 2. f3:**
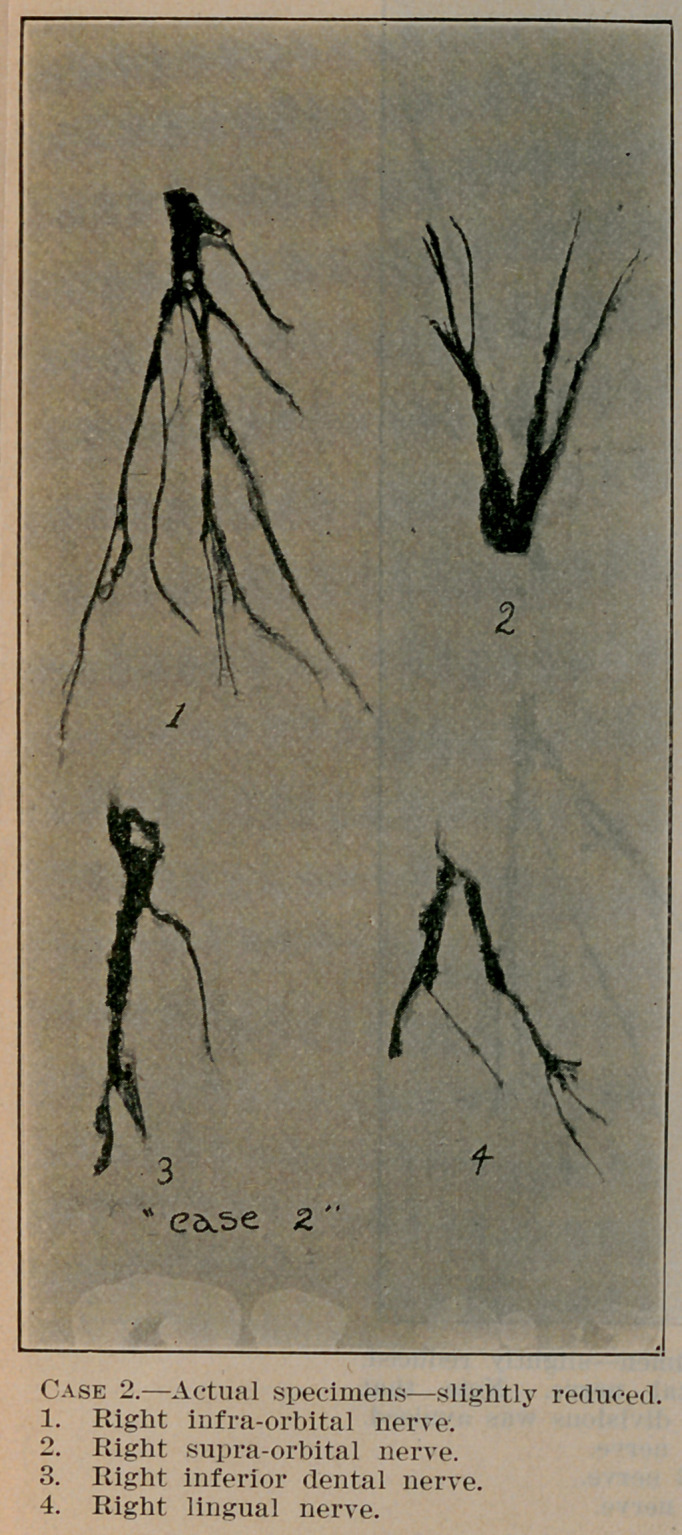


**Case 3. f4:**
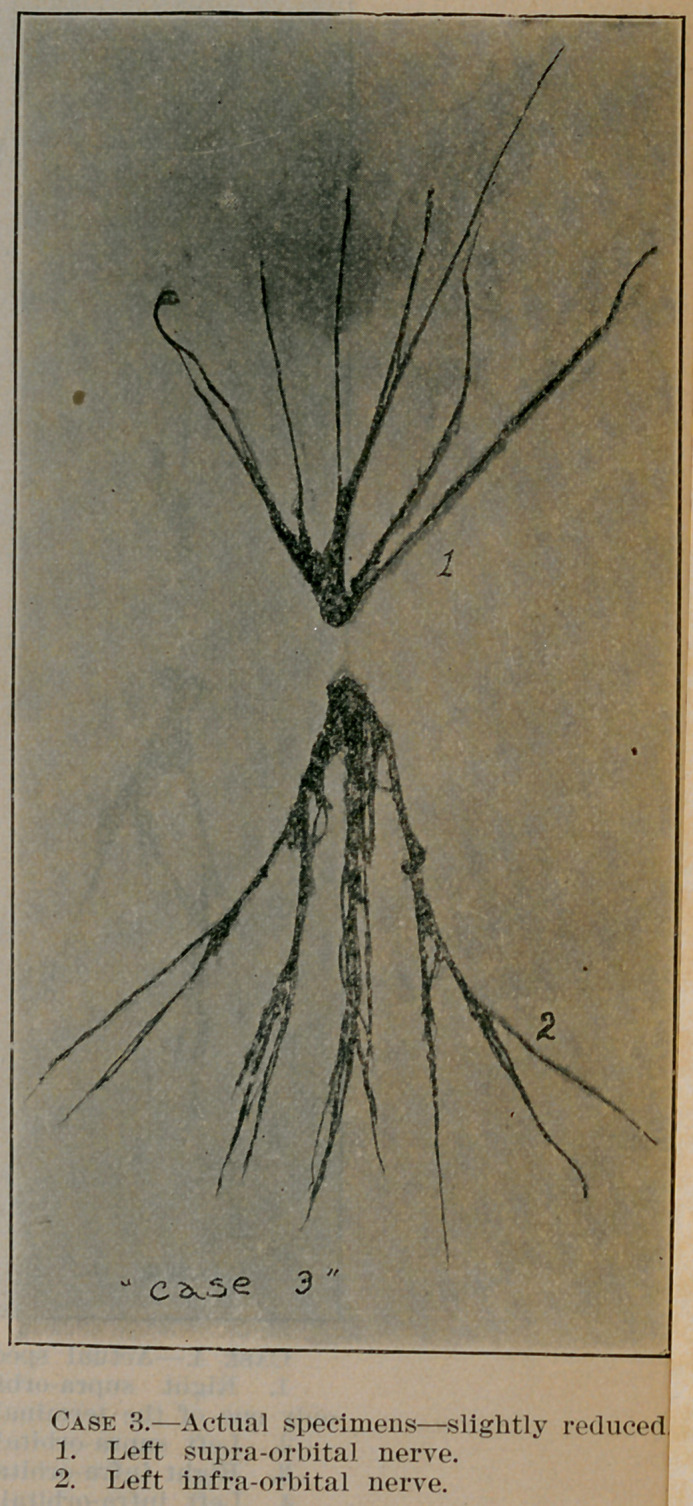


**Case 4. f5:**